# Supplementary material for: Sensors Network as an Added Value for the Characterization of Spatial and Temporal Air Quality Patterns at the Urban Scale
Source: Sensors (Basel). 2023 Feb 7;23(4):1859. doi: 10.3390/s23041859 (PMC9967040; doi:10.3390/s23041859)
Supplement: Supplementary file 1 [file sensors-23-01859-s001.zip › sensors-2102414-supplementary.pdf]

Supplementary material

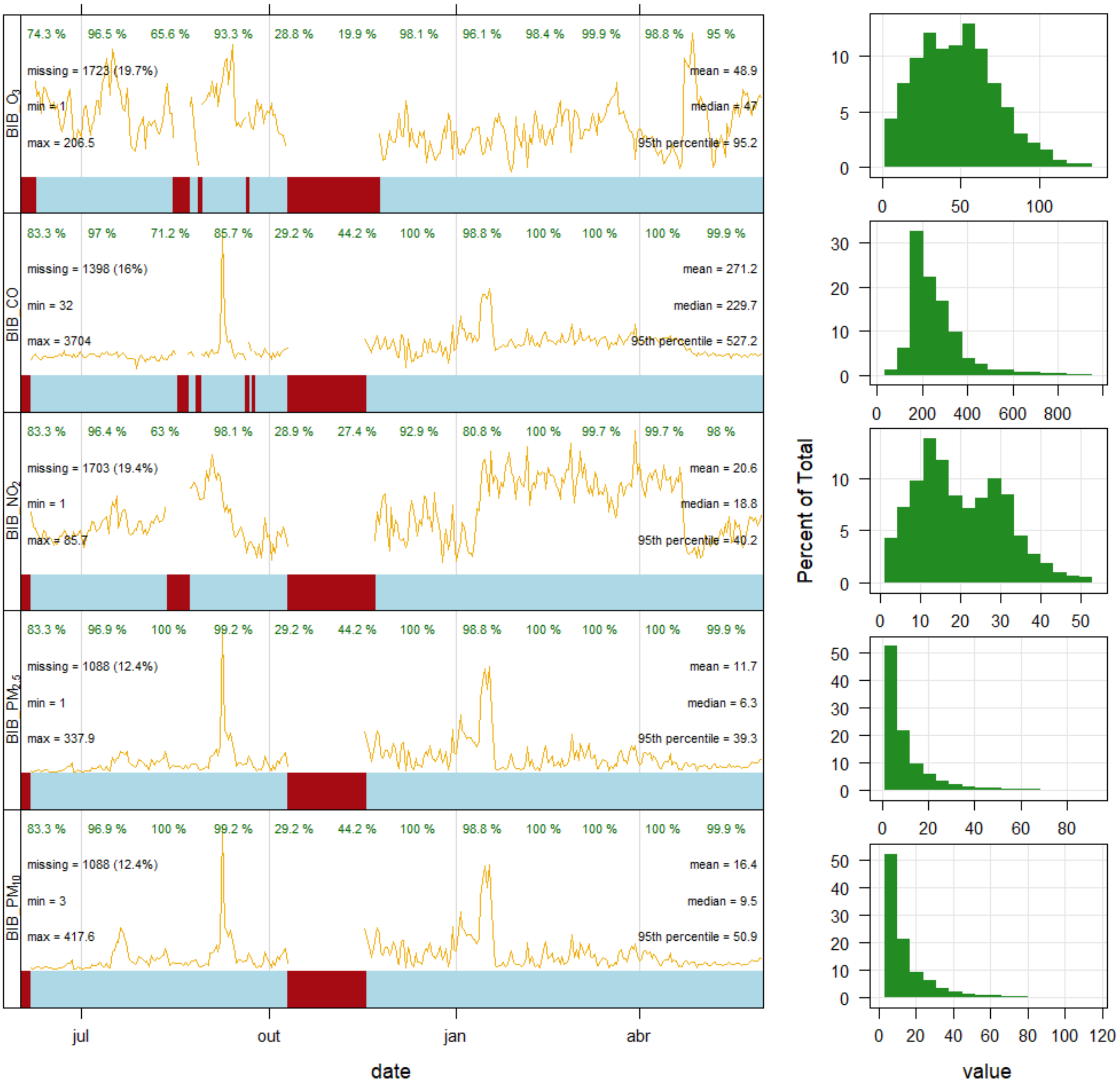

Figure S1. Timeseries of the daily means and density probability plot for all the pollutants measured in the Library sensors station.

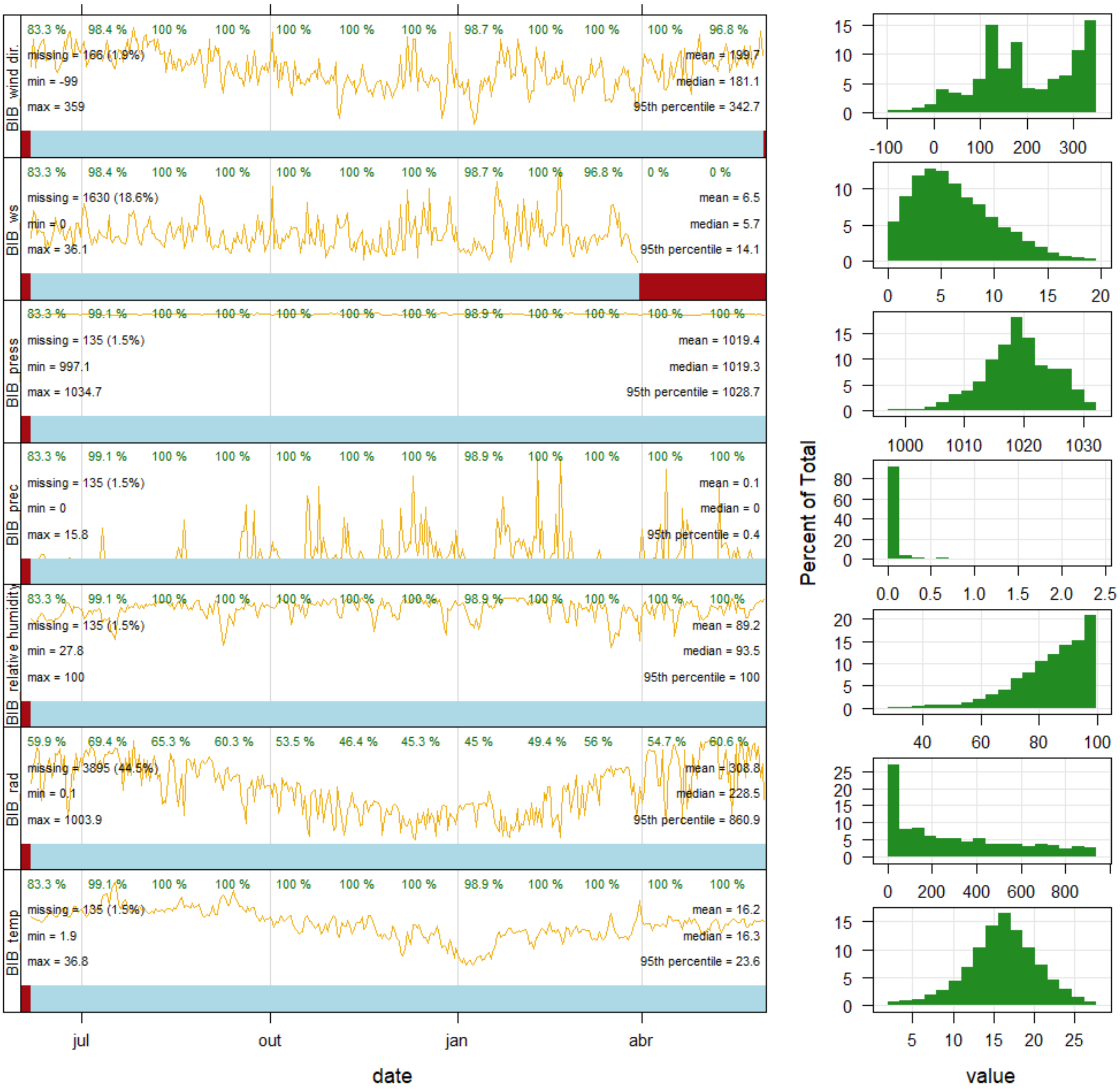

Figure S2. Timeseries of the daily means and density probability plot for all the meteorological parameters measured in the Library meteorological station.

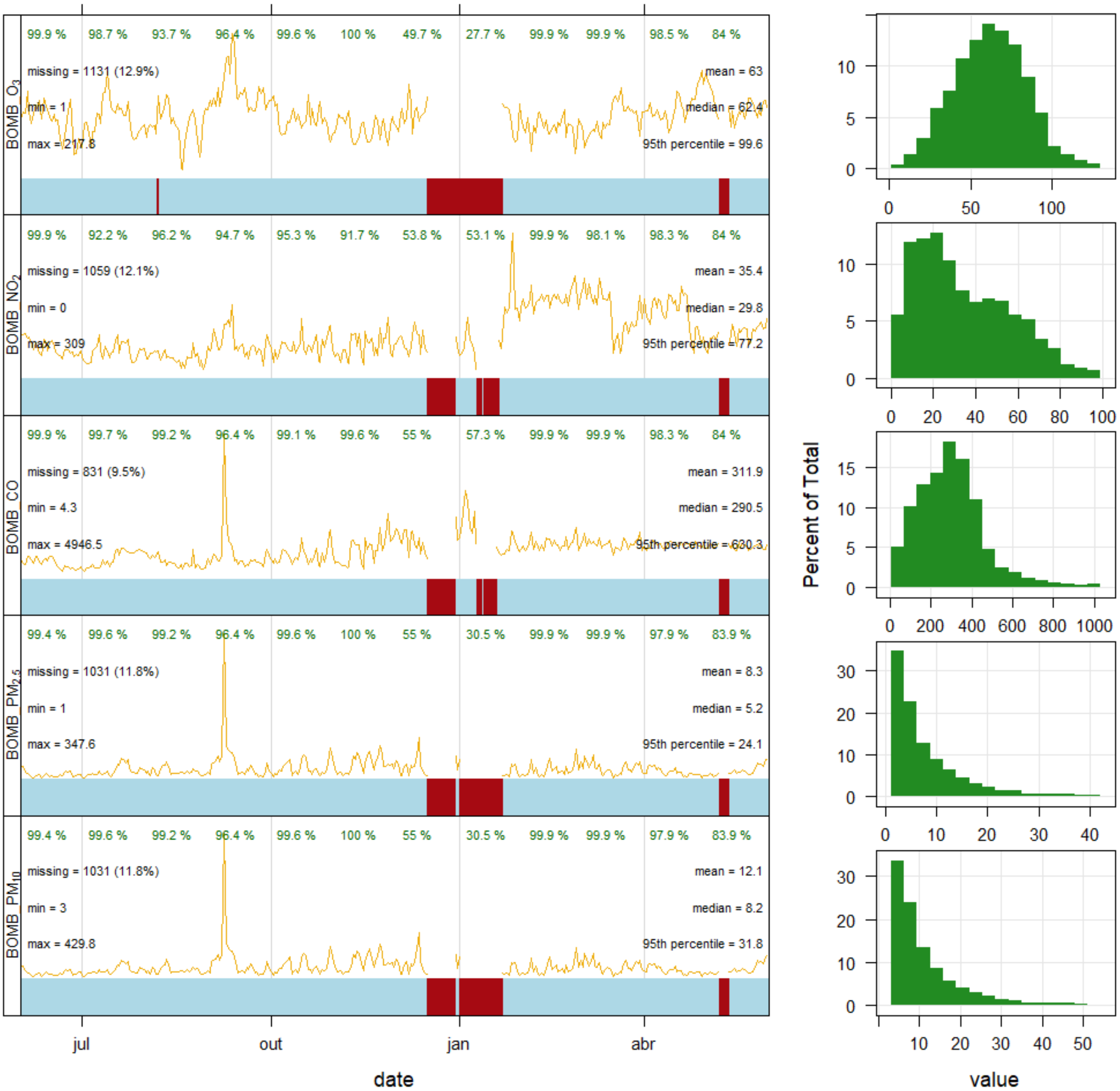

Figure S3. Timeseries of the daily means and density probability plot for all the pollutants measured in the Firefighters Headquarters sensors station.

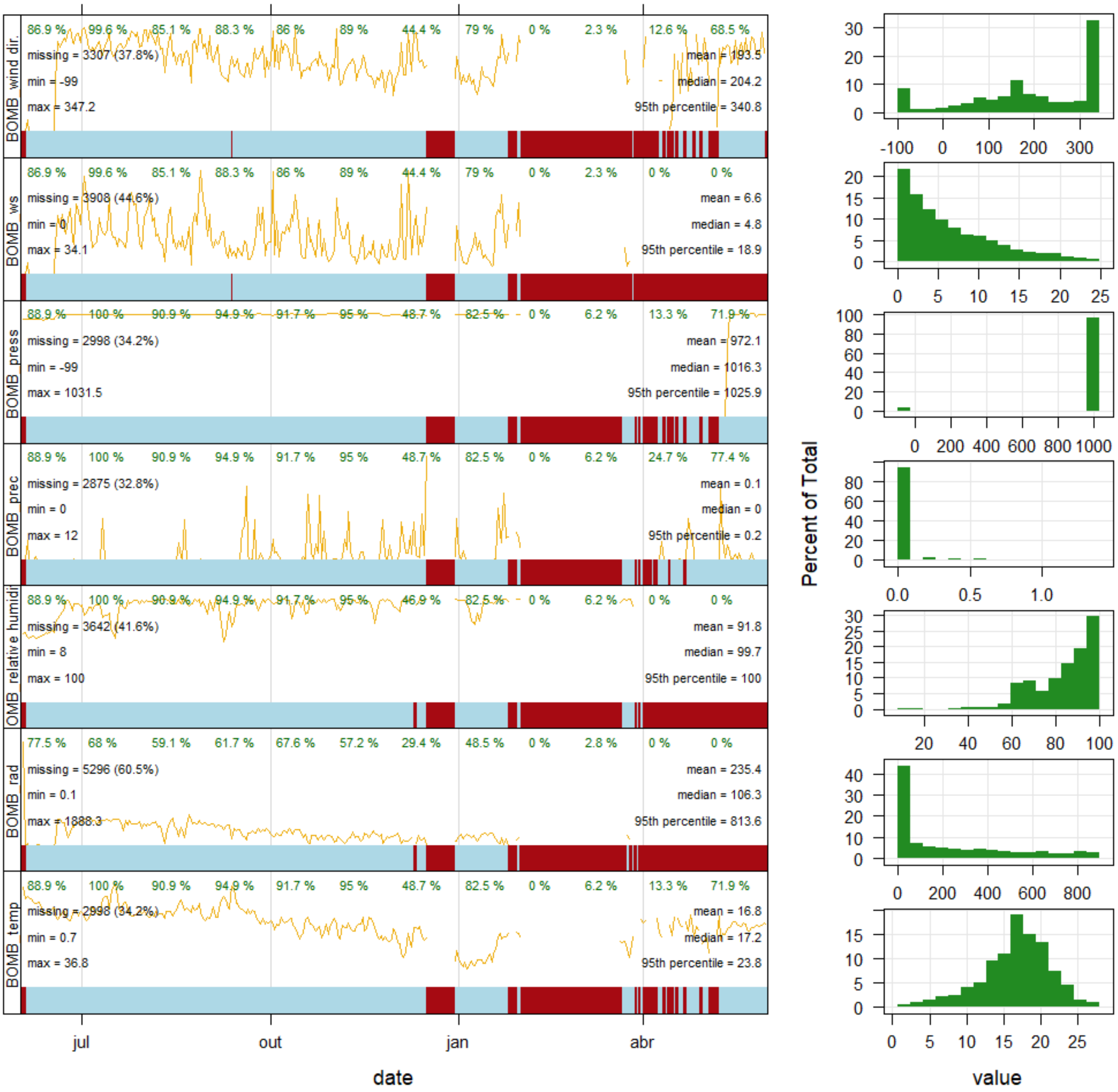

Figure S4. Timeseries of the daily means and density probability plot for all the meteorological parameters measured in the Firefighters Headquarters meteorological station.

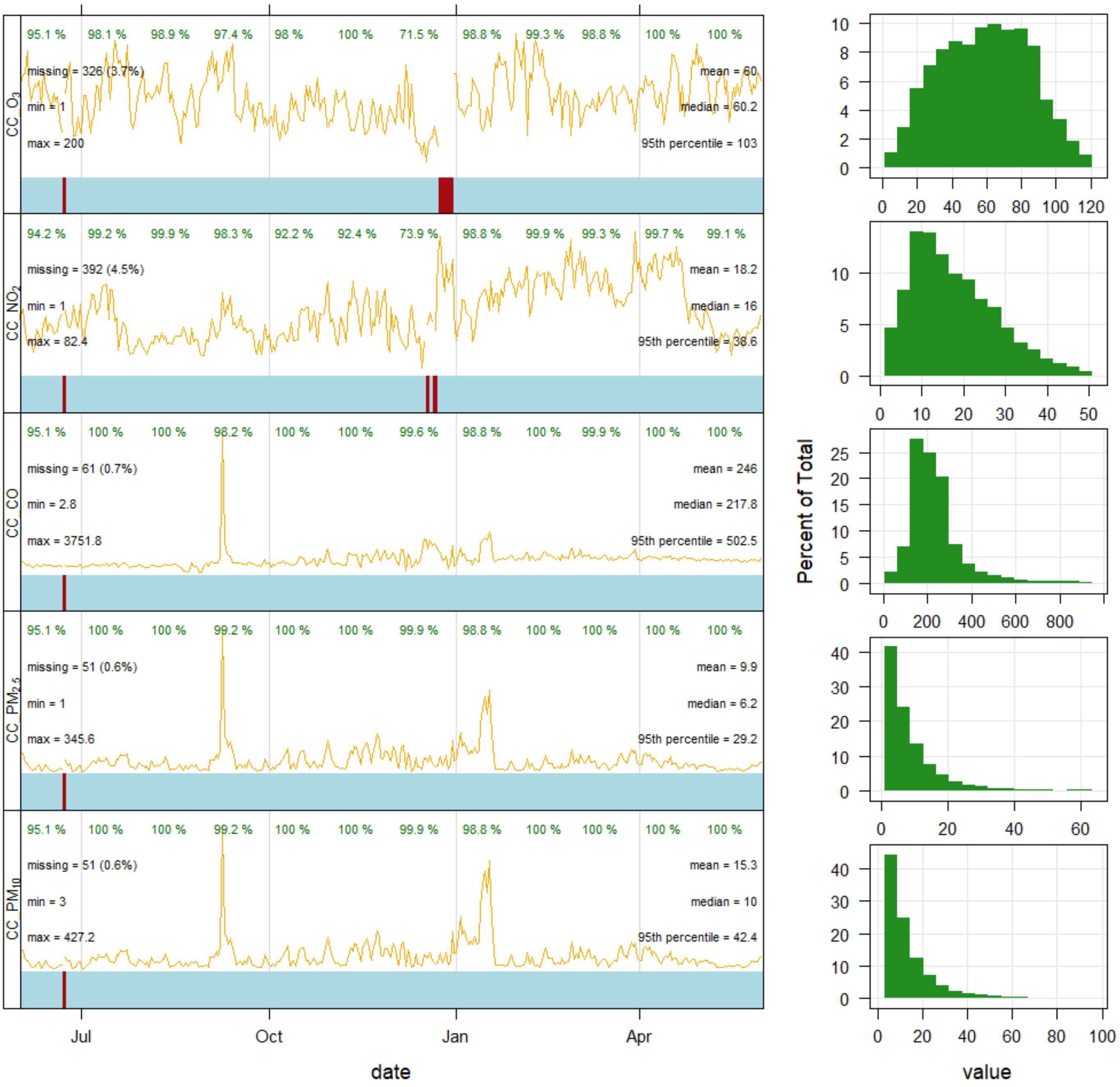

Figure S5. Timeseries of the daily means and density probability plot for all the pollutants measured in the Congress Centre sensors station.

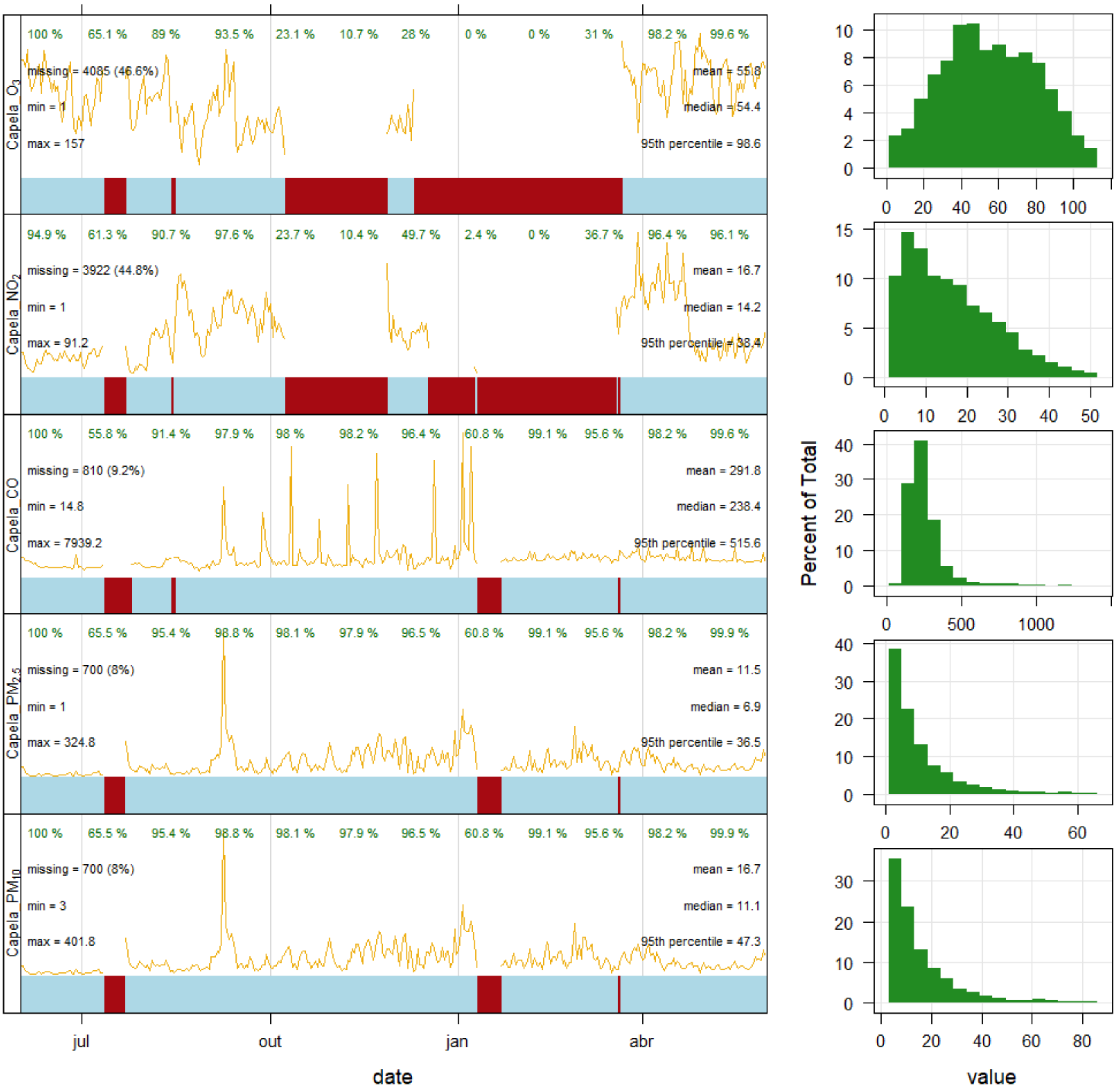

Figure S6. Timeseries of the daily means and density probability plot for all the pollutants measured in the Chapel sensors station.

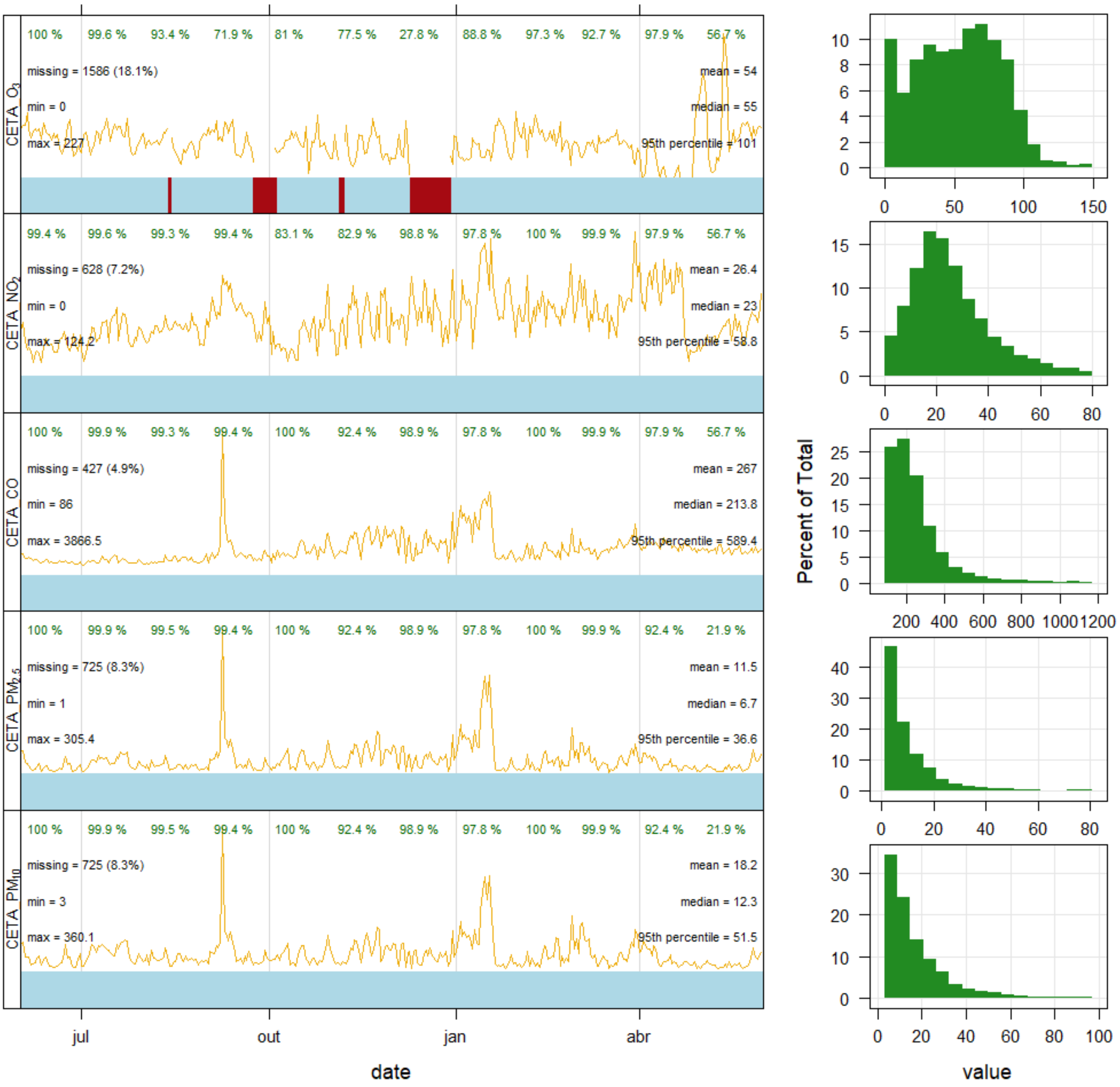

Figure S7. Timeseries of the daily means and density probability plot for all the pollutants measured in the CETA sensors station.

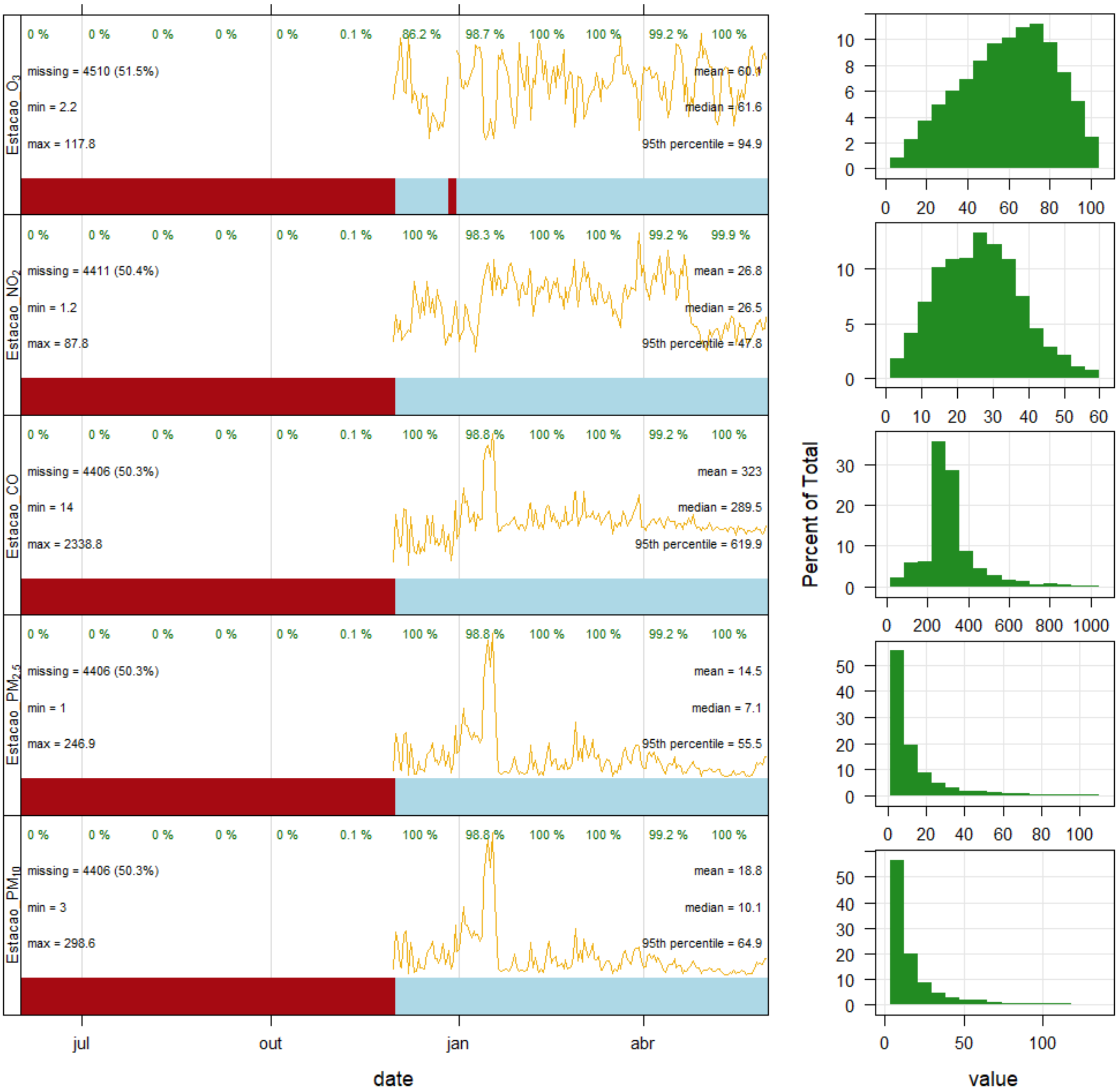

Figure S8. Timeseries of the daily means and density probability plot for all the pollutants measured in the Train Station sensors station.

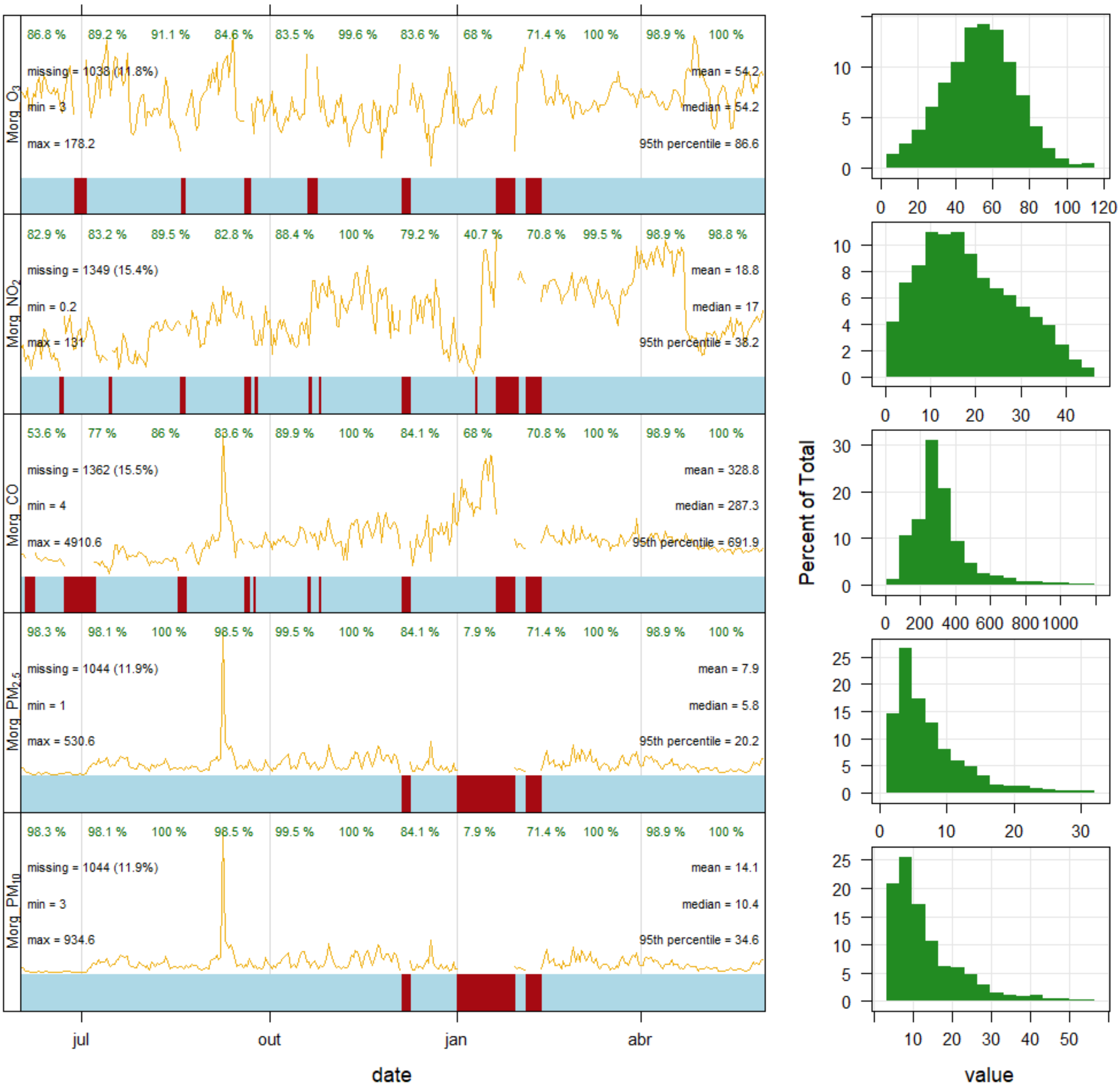

Figure S9. Timeseries of the daily means and density probability plot for all the pollutants measured in Morgados sensors station.

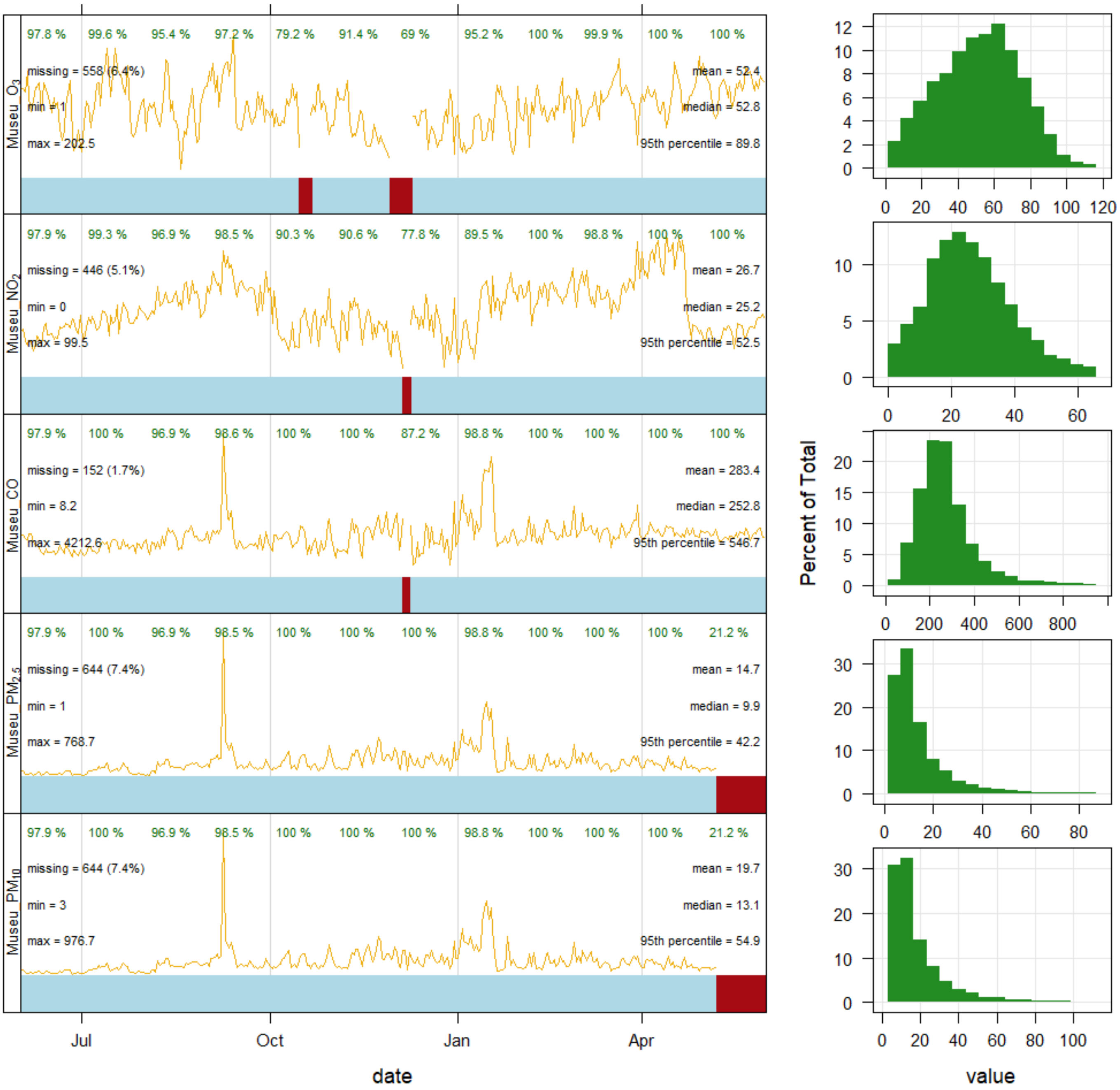

Figure S10. Timeseries of the daily means and density probability plot for all the pollutants measured in the Museum sensors station

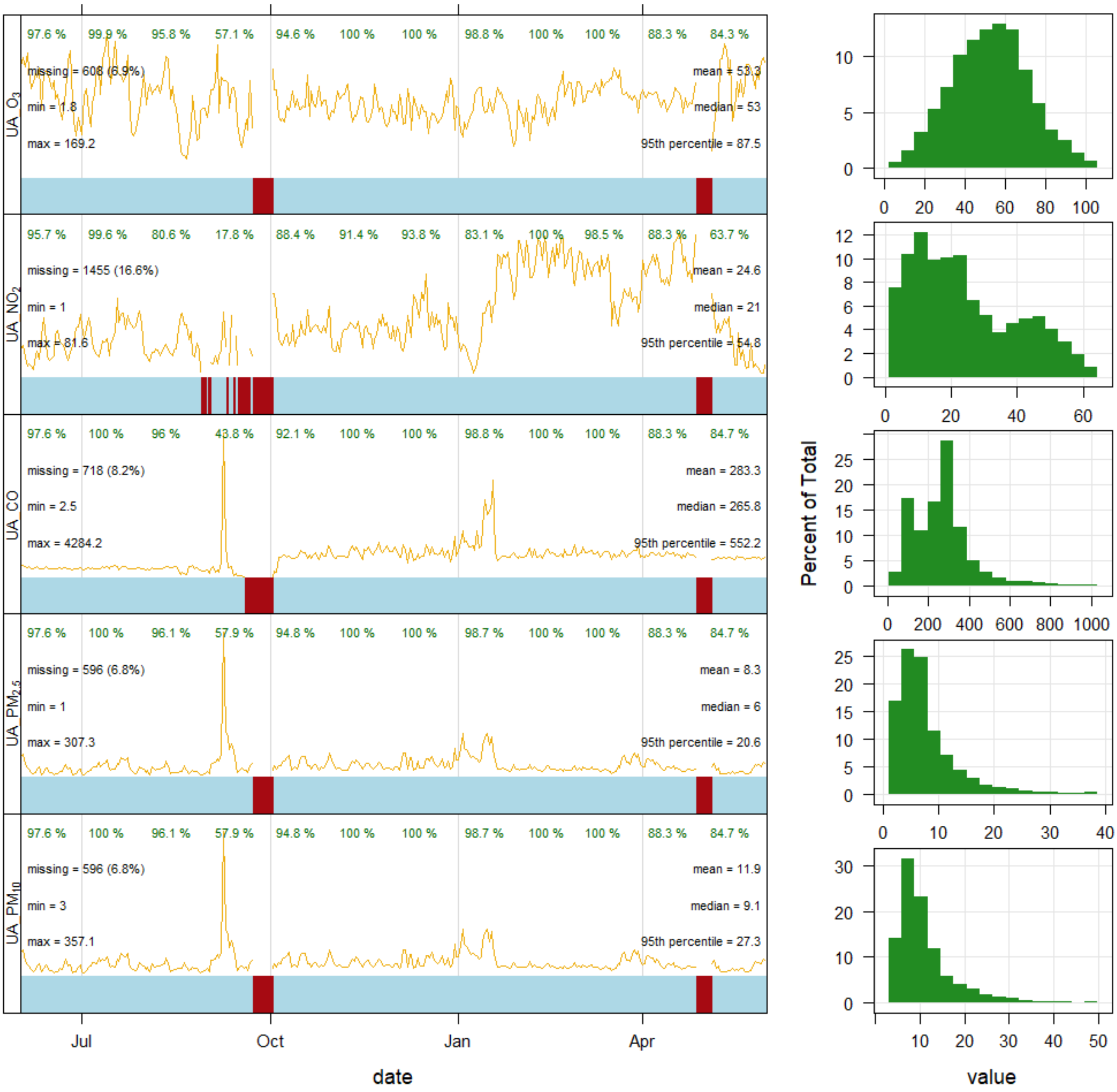

Figure S11. Timeseries of the daily means and density probability plot for all the pollutants measured in University sensors station

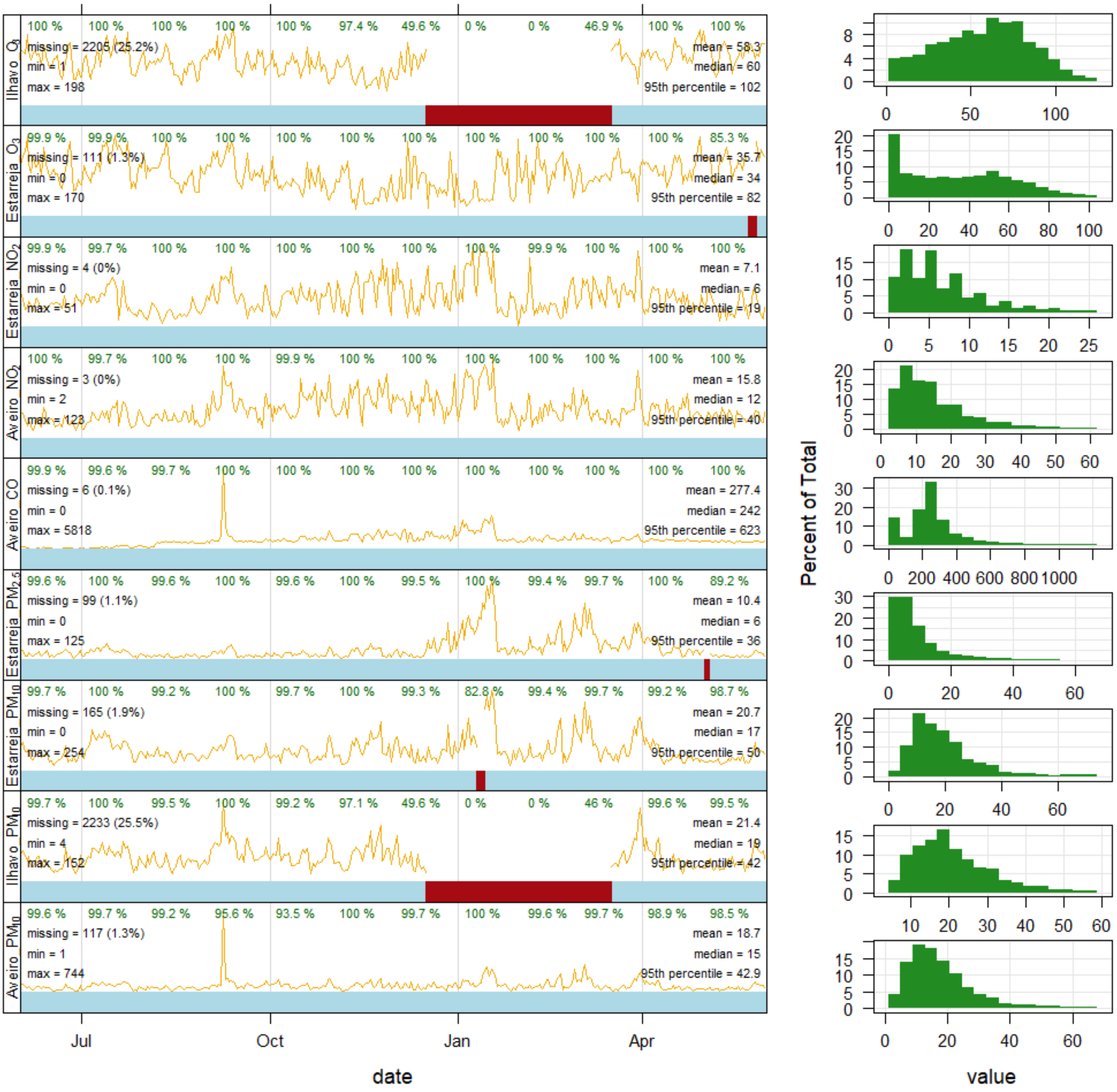

Figure S12. Timeseries of the daily means and density probability plot for PM10, PM2.5, NO_2 and O_3 in the reference stations of Aveiro, Estarreja and Ílhavo.

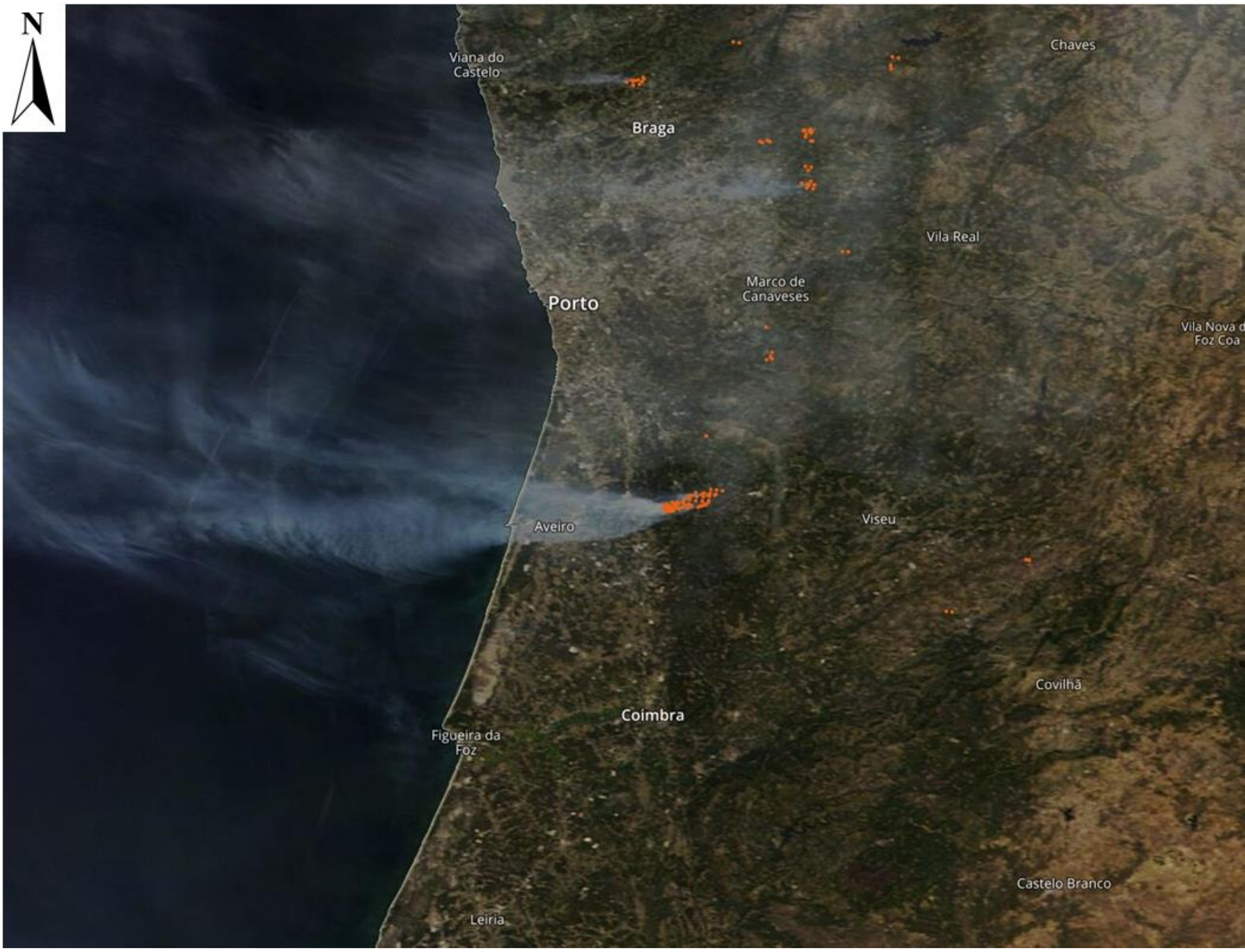

Figure S13. Satellite image of 8 September of 2020 as an example of one of the wildfires that occurred in September (WorldView, NASA).
